# Implantable Subcutaneous Peripheral Nerve Stimulation Improves Degenerative Ataxia

**DOI:** 10.7759/cureus.36991

**Published:** 2023-04-01

**Authors:** Athanasia Alexoudi, Eustathios Vlachakis, Spyros N Deftereos, Stefanos Korfias, Stylianos Gatzonis

**Affiliations:** 1 Department of Neurosurgery, National and Kapodistrian University of Athens, Evangelismos Hospital, Athens, GRC; 2 Department of Neurology, Neurology Practice, Athens, GRC

**Keywords:** implantable subcutaneous direct current stimulation, cerebellar ataxia, peripheral nerve stimulation, neuromodulation, movement disorder rehabilitation, degenerative ataxia

## Abstract

Degenerative cerebellar ataxias have no pharmacological or rehabilitation evidence-based treatment so far. Patients remain highly symptomatic and disabled despite receiving the best medical treatment available. This study investigates the clinical and neurophysiologic outcomes of the use of subcutaneous cortex stimulation (in keeping with the established protocol of peripheral nerve stimulation applied in chronic intractable pain) in degenerative ataxia.

We report a case of a 37-year-old right-handed man who developed moderate degenerative cerebellar ataxia at the age of 18 years. His symptoms progressively worsened and impaired his daily activities. We observed clinical improvement for at least one month following an initial two-week trial of parietal transcranial direct current stimulation. Although preoperative non-invasive transcranial neuromodulation application does not predict invasive cortex stimulation outcome, we pursued a long-lasting effect by implanting parietal and occipital subcutaneous electrodes.

At 12 months following permanent implantation, the patient exhibited amelioration of his symptoms and a change in neurophysiologic parameters.

Central neuromodulation based on peripheral stimulation is considered part of neurosurgical clinical practice for the treatment of a variety of neurological disorders. The underpinning neurophysiological mechanism that explains the effectiveness of the method has not been fully elucidated. We believe that further studies are warranted to investigate these promising results in such devastating conditions.

## Introduction

Degenerative cerebellar ataxias have no radical pharmacological or rehabilitation evidence-based treatment so far, and patients remain highly symptomatic and disabled despite receiving the best medical treatment available [1]. The cerebellum, on the other hand, has emerged as an attractive and promising target for neuromodulation in ataxias, as cerebellar areas present several connections with important cortical and subcortical structures, including the primary motor cortex (M1), the supplementary motor area, the cingulate cortex, and the basal ganglia. Transcranial direct current stimulation (tDCS), in particular, has shown promising results in several published studies [2].

We report the clinical and neurophysiologic findings in a patient with progressive degenerative ataxia in whom we observed clinical improvement for at least one month following an initial two-week trial of parietal tDCS. Although preoperative non-invasive transcranial neuromodulation application does not predict invasive cortex stimulation outcome, we pursued a long-lasting effect by implanting parietal and occipital subcutaneous electrodes [3]. At 12 months following permanent implantation, the patient exhibited amelioration of his symptoms and a change in neurophysiologic parameters. These initial results of the research project on the use of subcutaneous parieto-occipital direct current stimulation in ataxia are the first to be reported as far as we know.

## Case presentation

**Figure 1:**
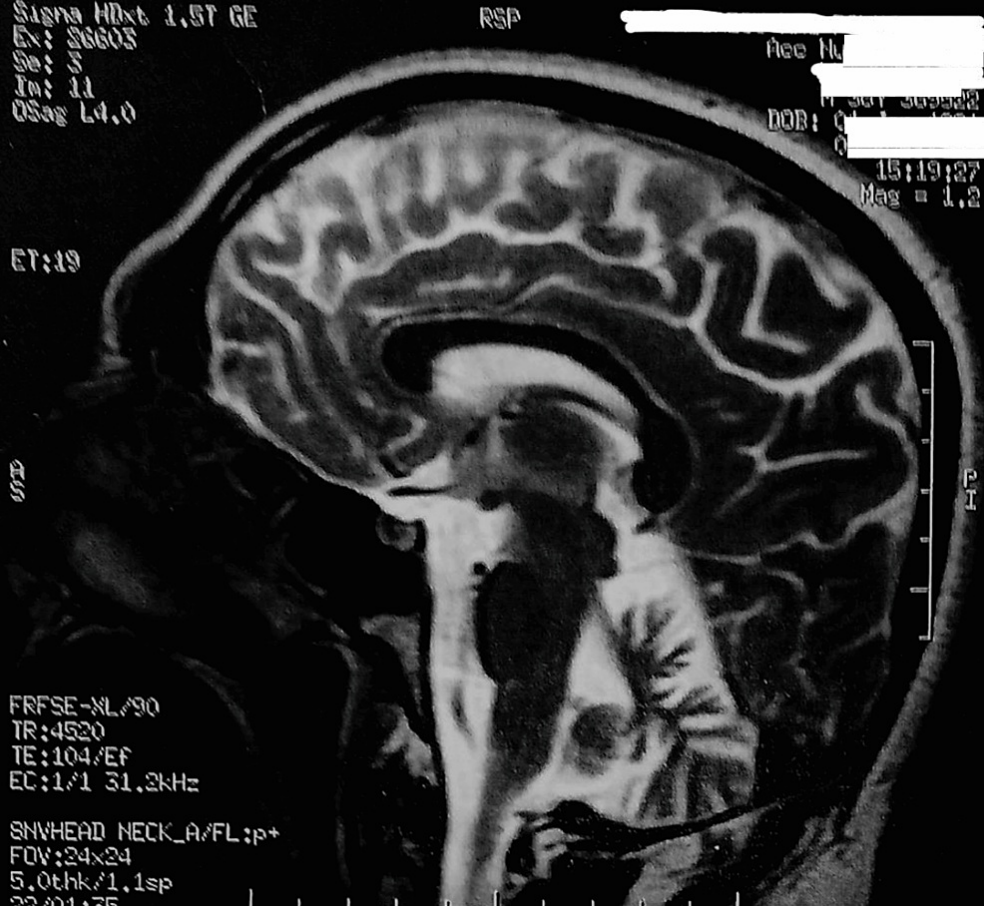
T2-weighted sagittal MRI showing moderate cerebellum atrophy.

A 37-year-old right-handed man developed moderate ataxia at the age of 18 years. His symptoms progressively worsened and significantly impaired his daily activities at the­ time of examination. Neurological examinations revealed normal muscle tone and intact deep tendon reflexes in all four extremities. There was no bradykinesia. Among the tests for coordination, the finger-nose and heel-to-shin tests were dysmetric on both sides. The patient had intentional tremors and dysdiadochokinesia and was unsteady upon standing up. He could not perform tandem gait and Romberg's sign was positive. His speech was dysarthric, and hypermetric saccades were observed on ocular examination. Sensory examination for pain, pressure, and proprioceptive sensation was normal. There was no family history of cerebellar ataxia or other neurodegenerative condition. Genetic testing for spinocerebellar atrophy (spinocerebellar ataxia (SCA) 1, SCA2, SCA3, SCA6, and SCA7) and Friedreich ataxia was negative. Brain magnetic resonance imaging revealed cerebellar atrophy (Figure 1); hence, a diagnosis of cerebellar ataxia was established. Although there is considerable discussion on the clinical meaningfulness of physician-rated scores in the ataxia field, at baseline, the Fahn-Tolosa-Marin Tremor Rating Scale (FTMRS) score was 31/144 and that of the Scale for the Assessment and Rating of Ataxia (SARA) was 14/40, indicating moderate dependence. His functional status score was 65 on the Functional Independence Measure and Functional Assessment Measure (FIM & FAM) [4-6].

He was treated with physical therapy and medication (amantadine, trihexyphenidyl, gabapentin, clonazepam, and acetazolamide) without satisfactory improvement. Considering the refractoriness of his symptoms and in accordance with published literature, we conducted a trial of 10 sessions (2 mA delivered for 30 minutes) of anodal tDCS over the left parietal cortex (Sooma tDCS^TM^, Sooma Medical, Helsinki, Finland) [2]. Cathode was positioned over the right mastoid. Previous trials in patients with degenerative cerebellar ataxias used either motor cortex and cerebellar anodal tDCS or cathodal spinal tDCS with equivocal results [7]. We chose to stimulate the parietal cortex considering that the primary somatosensory cortex expresses several forms of synaptic plasticity [8].

Clinical evaluations were performed on day zero (baseline) and one day after the 10th session. This resulted in significant improvement in ataxia that persisted for a period of a month; however, the patient eventually returned to his prior condition.

**Figure 2:**
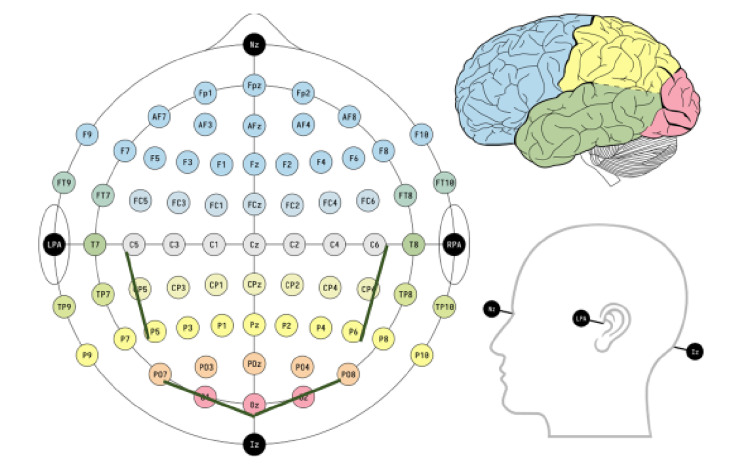
Positions of the four electrodes in the 10-10 system using modified combinational nomenclature.

Cortex-invasive stimulation neuromodulation has been used to treat a variety of neurological disorders such as refractory neuropathic pain, movement disorders, epilepsy, and tinnitus [9]. We chose to apply the technique toward improving the manifestation of ataxia. The patient underwent a trial of subcutaneous peripheral nerve stimulation (PNS) in keeping with the established protocol of PNS applied in chronic intractable pain [10]. During a two-week trial, four electrodes with eight contacts (Spectra™ System, Boston Scientific Corporation, Marlborough, MA) were implanted subcutaneously: two at the scalp over the parietal lobes and two at the scalp over occipital lobes (occipital nerve stimulation, PNS), under the superior nuchal line. The direction was from the midline under the external occipital protuberance to the apex of the mastoid process (Figure 2).

**Figure 3:**
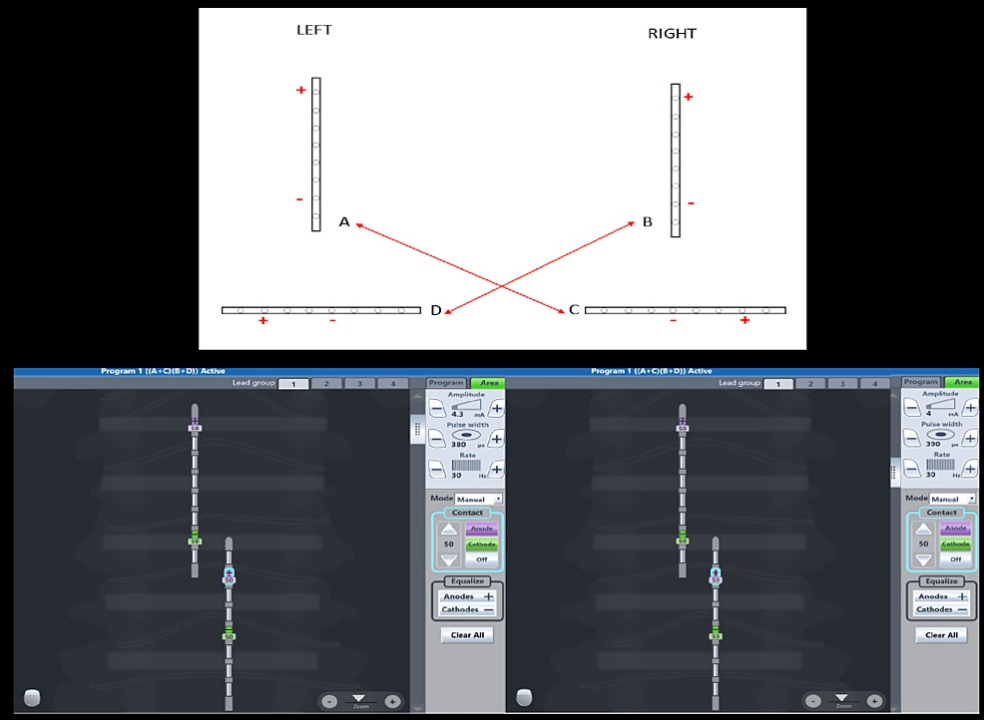
The stimulation parameters setting: 4.3 mA, 380 ms pulse width, and at 30 Hz in group A-C and 4 mA, 390 ms, and at 30 Hz in group B-D, respectively.

We observed an improvement in tremor (FTMRS) from 31/144 to 25/144 and ataxia (SARA) from 14/40 to 11/40; hence, permanent electrodes were then placed at the same positions six months after the trial. The most effective stimulation parameter settings are shown in Figure 3.

A blinded movement disorder specialist performed the clinical assessment and video recording using validated tools on five occasions: at baseline, at the trial, one day after the implantation (on and off conditions), six months (on condition) postoperatively, and one year (on condition) postoperatively. A neurophysiological evaluation by transcranial magnetic stimulation (TMS) was performed at the same timepoints, comprising measurement of the resting motor threshold (MT), and cortical silent period (CSP).

The study has been approved by the ethics committee of the National and Kapodistrian University of Athens. We obtained informed consent from the patient prior to all procedures.

We observed an improvement in FTMRS and SARA in the on-stimulation condition one day after the implantation, six months postoperatively, and one year postoperatively compared to baseline. The score evaluating his functional status increased from 65 to 95 in FIM & FAM and he described the change on the Patient Global Impression of Change (PGIC) scale as 5. There was a re-emergence of ataxia (from 31/144 to 35/144 in FTMRS and from 14/40 to 15/40 in SARA) when the stimulator was deactivated one day after the implantation (Table 1).

**Table 1:** The Fahn-Tolosa-Marin Tremor Rating Scale (FTMRS) and the Scale for the Assessment and Rating of Ataxia (SARA) scores at baseline, at the trial, one day after the implantation (on and off conditions), six months (on condition) postoperatively, and one year (on condition) postoperatively. * SARA reduction in the on-stimulation condition at one day after the implantation, six months postoperatively, and one year postoperatively compared to baseline. ** FTMRS-TOTAL reduction in the on-stimulation condition at one day after the implantation, six months postoperatively, and one year postoperatively compared to baseline.

	Preoperative	Trial	Postoperative			
	Baseline	Trial	1 day	1 day	6 months	1 year
		ON	ON	OFF	ON	ON
SARA total	14	11	11 (21.4%)*	15	10.5 (25%)*	10 (28.6%)*
FTMRS - A	9	5	5	8	5	5
FTMRS - B	8	10	6	11	9	10
FTMRS - C	14	12	11	16	11	9
FTMRS - total	31	27	22 (28.1%)**	35	25 (19.4%)**	24 (22.6%)**

MT was slightly reduced on the right one day postoperatively but returned to baseline levels thereafter, while on the left, it remained stable at all timepoints. CSP remained stable when stimulating the left cortex (recording at the right limbs); however, when stimulating the right cortex (recording at the left limbs), it decreased sizably at all timepoints. Interestingly, on postoperative day one, when CSP was recorded at the on- and off-stimulation conditions, it remained decreased at the on-condition but roughly doubled at the off-condition (Table 2). No side effects were observed.

**Table 2:** The neurophysiological evaluation by transcranial magnetic stimulation (TMS) at five timepoints comprising measurement of the resting motor threshold (MT) and cortical silent period (CSP). * Falsely high value.

	Recording at the right limbs						Recording at the left limbs					
	Baseline	Trial	Postoperative 1 day	Postoperative 1 day	Postoperative 6 months	Postoperative 1 year	Baseline	Trial	Postoperative 1 day	Postoperative 1 day	Postoperative 6 months	Postoperative 1 year
		ON	ON	OFF	ON	ON		ON	ON	OFF	ON	ON
Motor threshold (%)	55	40	45	47	55	50	55	55	50	60	55	55
Cortical silent period (ms)	236	238	243	278	241	262	999*	245	264	412	251	252

## Discussion

Cerebral synaptic inputs to cerebellar neurons and output nuclei have connections with the prefrontal, parietal, and sensory cortexes as well as the motor and premotor cortex [11]. The cerebellum receives input from the cerebral cortex and projects to the motor and premotor cortices and the ventrolateral nucleus of the thalamus via the dentate nucleus (DN). It has been proposed that sensory processing, and not motor control, is the main function of cerebellar sensorimotor circuits [12]. In patients with spinocerebellar ataxia, motor adaption is associated with grey matter volume loss in the lateral cerebellum and the inferior parietal lobule [13]. Through the inhibition of the DN, the cerebellum exerts its inhibitory effect on the motor cortex, toward the control of motor activity. CSP is prolonged in neurodegenerative cerebellar ataxias, reflecting reduced cerebellar brain inhibition [14]. It seems that anodal cerebellar tDCS, which stimulates deeper structures of the brain, increases the inhibition, restoring physiological cerebellar brain inhibition pathways [15,16]. However, it remains unclear what type of modulation (if any) occurs in cerebellar circuits during the PNS application.

In the stimulation-off condition, we noticed a deterioration of symptoms (compared with the baseline visit), as it is reflected in FTMRS and SARA, allowing us to assume a disease progression. Nevertheless, during the last follow-up visit (20 months after the baseline visit), we obtained the most impressive reduction in SARA, surmounting the degenerative nature of the disease. Considering that the minimal clinically important worsening on the SARA in the most common ataxias ranges from 0.81 to 1.83 points, corresponding to the mean annual decline, our pivotal case illustrates that subcutaneous parietal stimulation can exert a long-lasting beneficial clinical effect in cerebellar ataxia [17].

Another possible mechanism lies in the role of the cerebellum and the parietal cortex in motor adaptation. Computational studies suggest that the function of the cerebellum is to create a model that predicts the sensory outcome system of motor commands through internal connections and feedback. The integration of the predicted proprioceptive and visual outcomes with sensory feedback is the output of the model, connected to parietal cortex function. The parietal cortex transfers the sensory prediction error signals to the cerebellum to modify the model. In this way, the inhibitory output of Purkinje cells is regulated toward reducing errors in movements [18]. Cortical excitability may be affected by PNS [19]. The reduction of CSP on the more affected side raises the question: Is the restoration of inhibitory cerebellar output implicated in the underlying neurophysiological mechanism?

We acknowledge that we present a single case report and not a double-blind cross-over trial. Another limitation is that the patient was aware that he received real tDCS and PNS, and placebo response besides depression should be considered when designing and interpreting clinical trials in cerebellar ataxia patients [20].

## Conclusions

Central neuromodulation based on peripheral stimulation is considered part of neurosurgical clinical practice for the treatment of a variety of neurological disorders. The underpinning neurophysiological mechanism that explains the effectiveness of the method has not been fully elucidated. We believe that further studies are warranted to investigate these promising results in such devastating conditions.
